# A systematic review and bibliometric study of Bertolotti’s syndrome: clinical characteristics and global trends

**DOI:** 10.1097/JS9.0000000000000541

**Published:** 2023-06-14

**Authors:** Wenhao Zhu, Xing Ding, Jiale Zheng, Fan Zeng, Fan Zhang, Xuequn Wu, Yijun Sun, Junming Ma, Mengchen Yin

**Affiliations:** a Longhua Hospital, Shanghai University of Traditional Chinese Medicine; b Shanghai Jiao Tong University Affiliated Sixth People’s Hospital; c Postdoctoral Station, Changzheng Hospital, Second Affiliated Hospital of Naval Medical University, Shanghai, China

**Keywords:** bertolotti’s syndrome, bibliometric analysis, characteristics, research development, systematic review

## Abstract

**Purpose:**

Bertolotti’s syndrome is a prevalent congenital deformity. However, many physicians fail to include it in their differential diagnosis for low back pain (LBP), which results in missed diagnosis or misdiagnosis. There is still a lack of standardized treatment and management strategies for Bertolotti’s syndrome. This study aimed to review the clinical characteristics and management of Bertolotti’s syndrome and reports bibliometric insights in advancements in Bertolotti’s syndrome research.

**Methods:**

Studies published until 30 September 2022 were systematically reviewed according to the PRISMA guidelines. Three independent reviewers extracted the data and assessed the quality and risk of bias of the studies based on the methodological index of non-randomized studies (MINORS). SPSS, VOS viewer, and the Citespace software were used for the systematic review, visual analysis, data mining, mapping, and clustering of the retrieved articles, which presented clear and visual presentations of the structural patterns of published research in graphs.

**Result:**

A total of 118 articles, describing a total of 419 patients with Bertolotti’s syndrome, were included. There was an upward trend with a steady increase in the number of publications. The world map distribution showed that most publications were predominantly from North America and Asia. The most cited articles were published in the following journals: Spine, J Bone Joint Surg, and Radiology. The mean age of the patients was 47.7 years, and 49.6% of them were male. A total of 159 (96.4%) patients had LBP symptoms. The mean symptom duration was 41.4 months (74.8%), and most of the patients had Castellvi type II. Disc degeneration was the most reported comorbid spinal diseases. The mean methodological index of non-randomized studies score was 4.16±3.95 points (range, 1–21). A total of 265 (68.3%) patients underwent surgical treatments. Minimally invasive surgical techniques, prevalence, image classification, and disc degeneration were the current main research areas of Bertolotti’s syndrome.

**Conclusions:**

The steady increase in the number of publications demonstrated the increased attention of researchers on this topic. Our results showed a significant prevalence of Bertolotti’s syndrome in patients with LBP and a long symptom duration before the initiation of treatment. Surgical treatments were commonly used to treat patients with Bertolotti’s syndrome after a non-effective conservative treatment. Minimally invasive surgical techniques, prevalence, image classification, and disc degeneration are the major research areas of Bertolotti’s syndrome.

## Introduction

Highlights

- This study firstly reviews the clinical characteristics and management of Bertolotti’s syndrome.
- We conducted a comprehensive and objective search of articles published to obtain reliable results and provide some insights into its clinical characteristics, overall knowledge structure, and global trends in research development.
- Minimally invasive surgical techniques, prevalence, image classification, and disc degeneration are major research areas of Bertolotti’s syndrome.

Patients with chronic pain or functional impairment secondary to lumbosacral transitional vertebrae (LSTV) are diagnosed with Bertolotti’s syndrome, one of the most common congenital deformities of the lumbosacral vertebrae^1–8^. It was first reported by Mario Bertolotti’s in 1917. The shape of the sacral lumbar vertebrae is varied, from hypertrophy of the transverse process to complete fusion of the transverse process and sacrum. In 1984, Castellvi determined the following four defining types of LSTV by examining the myelograms of 200 patients: type I—transverse process hypertrophy, measuring greater than 19 mm in width; type II—transverse process hypertrophy leading to an incomplete sacralization (L5) or lumbarization (S1); type III—complete transverse process sacralization or lumbarization; and type IV—mixed complete sacralization and incomplete sacralization^9^. In type II LSTV, “pseudoarticulation” of an enlarged transverse process with the sacral ala occurs. Nardo *et al.* ^10^ reported that 73% of patients with type II LSTV had low back pain (LBP). However, the mechanism by which LBP and other symptoms secondary to LSTV occurs remain unclear. The LSTV may change the biomechanical properties of the lumbosacral region, resulting in abnormal movement and asymmetric stress distribution in the lumbosacral region^11,12^. The enlarged transverse process could cause intervertebral foramen stenosis and, subsequently, nerve compression^13^.

The diagnosis of Bertolotti’s syndrome, a syndrome caused by LSTV, requires the combination of clinical evaluations and imaging investigations. Based on the literature, the incidence of this syndrome ranges widely from 4 to 35%. Due to its similarity with other diseases that also present as LBP, many patients may never be diagnosed accurately. In addition, the clinical symptoms of Bertolotti’s syndrome often correlate poorly with imaging findings, which makes radiological identification difficult^14^. Although Bertolotti’s syndrome is a prevalent congenital deformity, many physicians fail to include it in their differential diagnosis for LBP, resulting in missed or misdiagnosis^15–17^.

In the past, progressive stepwise treatments, which relieved patient symptoms while providing relevant information for diagnosis, were adopted in most cases. The treatments typically started with initial conservative treatments such as physical therapy and oral nonsteroidal analgesics and progress to interventional approaches, including steroid injections and surgical resection or fusion^18–20^. Although the available treatments are numerous, standardized treatment and management strategies for Bertolotti’s syndrome are still lacking. It is necessary to summarize the diagnosis and therapy for Bertolotti’s syndrome, which is a prevalent but neglected disease. In the current study, we conducted a comprehensive systematic review to investigate the clinical characteristics (symptoms, pathological type, and epidemiological information) and therapeutic measures of all Bertolotti’s syndrome cases reported in the literature. Hopefully, this study can aid clinicians in the assessment and diagnosis of Bertolotti’s syndrome and provide a reference for the selection of appropriate treatment strategies. To our knowledge, the present study is the first systematic review about the clinical characteristics and management of Bertolotti’s syndrome and the first to report research developments and bibliometric insights in Bertolotti’s syndrome.

## Material and methods

### Search strategy

The literature was systematically reviewed using the PRISMA (Reporting Items for Systematic Reviews and Meta-Analyses) guideline and assessed the methodological quality of systematic reviews using AMSTAR guidelines^21–23^. Firstly, we used a broad search terminology, including “Bertolotti’s syndrome” OR “lumbosacral transitional vertebrae” OR “far-out syndrome” OR “Richard disease” OR “Lateral exit zone stenosis,” without filtering. The PubMed, Embase, Medline, Scopus, and Cochrane Library databases were thoroughly searched to identify relevant studies that were published until 30 September 2022. In addition, three independent reviewers (Z.W.H., D.X., and Q.L.) screened all the titles and abstracts of the retrieved studies based on the eligibility criteria, and subsequently, the full texts of the eligible articles, which were in English, were reviewed to ensure that they fully met the inclusion criteria. The studies included case reports, clinical trials, case series, basic researches, animal testing, reviews, cadaveric studies, and imaging studies. Letters, editorial materials, papers, and corrections were excluded from the final retrieved studies. To ensure accurate inclusion of articles, two senior reviewers (Y.M.C. and M.J.M.) were consulted if there was a disagreement on the inclusion of a study among the three reviewers. Finally, a meeting was held to settle any disagreement on the inclusion of a study. The protocol of review has been registered in PROSPERO.

### Data extraction

After the final selection of the articles to be included in the study, the required data was extracted onto a customized sheet. Three reviewers independently extracted the corresponding data from each article, including the year of publication, publication country, keywords, number of cases, and patients’ demographic data (e.g. sex, age, and Castellvi types). Moreover, we described patients’ symptoms, symptom durations, comorbid diseases, and received treatments (e.g. operative or non-operative treatments).

### Quality assessment (risk of bias)

Three reviewers (D.X., Z.J.L., and Z.F.) independently assessed the quality and risk of bias of the selected studies based on the methodological index of non-randomized studies (MINORS)^24^. The MINORS tool consists of 8 items, each scored 0–2, with a maximum of 16 points for non-comparative studies and 24 points for comparative studies; higher MINORS scores indicate higher study quality and lower risk of bias.

### Statistical analysis

Categorical data are presented as frequencies and percentages and numerical data as means, ranges, and standard deviations. Furthermore, annual trends of publications, distribution, citation, co-authorship status, research hotspots, and co-citation status were quantitatively and qualitatively analyzed. VOS viewer and the Citespace software were used for visual analysis, data mining, mapping, and clustering of the retrieved articles, which provided clear and visual presentations of the structural patterns of published studies as graphs.

## Results

### Study identification

**Figure 1:**
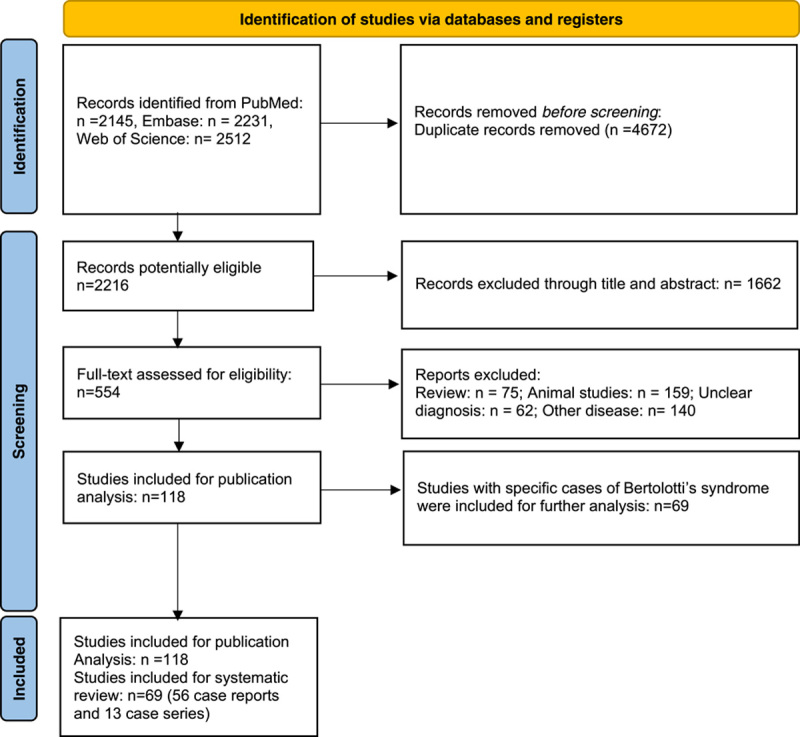
Study flow chart.

After excluding duplicate records, 2017 relevant studies remained, of which 540 were selected after screening the titles and abstracts, and their full texts were assessed independently by three authors (X.D., J.L.Z., and M.C.Y.). Based on the inclusion and exclusion criteria, 118 studies were finally included for analysis. The selection flow chart is shown in Figure 1. Among the 118 articles, Bertolotti’s syndrome was specifically reported in 69 studies, including 56 case reports and 13 case series, describing a total of 419 patients with Bertolotti’s syndrome.

**Figure 2:**
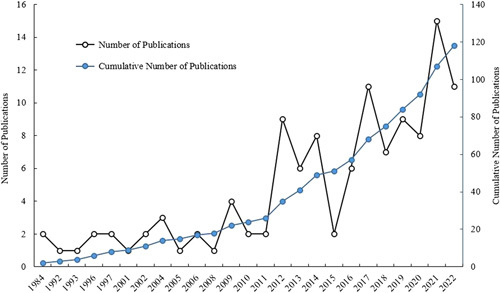
Annual trends in publications.

**Figure 3:**
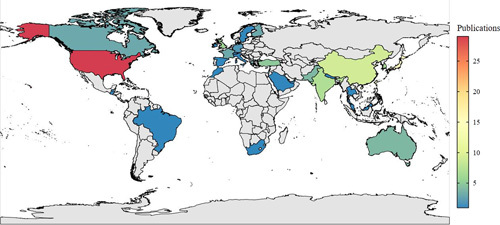
Worldwide map of research productivity.

Overall, there was an upward trend with a steady increase in the number of publications. A total of eight articles were published before the year 2000, and the increase occurred in the following years. The number of published studies peaked from the year 2017–2022. A total of 15 (12.7%) studies were published in 2021, the highest compared with all the years, followed by a total of 22 studies in the years 2017 and 2022. This result indicated that increasing efforts and explorations have been made on Bertolotti’s syndrome. Figure 2 shows the annual publications trends. The number of studies published in the USA accounted for 24.6% (29/118) of all the included studies, followed by Japan (16), China (9), India (7), and South Korea (6). The world map distribution showed that most of the publications were predominantly from North America and Asia (Fig. 3).

The included studies were published in 85 journals. Only 9 (7.6%) journals published more than 3 of the included studies. The journal with the greatest number of publications was Spine, with a total of 7 studies. The second ranked journal was Cureus J Med Sci with 4 studies, followed by Asian Spine J and Eur Spine J with 3 studies each. Table 1 shows the top 10 cited articles in terms of study title, journal, authors, year of publication, and citation numbers. The highest and lowest number of article citations were 273 and 57, respectively. Three of these top 10 cited articles were published in Spine, and 2 each were published in J Bone and Joint Surg and Radiology.

**Table 1:** Top 10 cited articles on bertolotti’s syndrome.

No.	Title	First author	Year	Journal name	Total times cited	Annual cited
1	Lumbosacral transitional vertebrae and their relationship with lumbar extradural defects	Castellvi^9^	1984	Spine	273	7.18
2	Lumbosacral Transitional Vertebrae: Classification, Imaging Findings, and Clinical Relevance	Konin^41^	2010	Am J Neuroradiol.	163	13.58
3	Lumbosacral transitional vertebra - Relation to disc degeneration and low back pain	Luoma *et al*.^11^	2004	Spine	142	7.89
4	Intervertebral disc degeneration associated with lumbosacral transitional vertebrae - A clinical and anatomical study	Aihara *et al*.^12^	2005	J Bone Joint Surg	101	5.94
5	Alar transverse process impingement of the L5 spinal nerve: the far-out syndrome	Wiltse^26^	1984	Spine	98	2.58
6	Bertolotti’s syndrome - A cause of back pain in young people	Quinlan^16^	2006	J Bone Joint Surg.	86	5.38
7	Lumbosacral Transitional Vertebrae: Association with Low Back Pain	Nardo *et al*.^10^	2012	Radiology	79	7.90
8	Verification of lumbosacral segments on MR images: identification of transitional vertebrae	Hahn^27^	1992	Radiology	66	2.20
9	Variations in morphology of the lumbosacral junction on sagittal MRI: Correlation with plain radiography	O’Driscoll^37^	1996	Skeletal Radiol	62	2.38
10	Surgical treatment of Bertolotti’s syndrome. Follow-up of 16 patients	Santavirta *et al*.^36^	1993	Arch Orthop Trauma Surg	57	1.97

### Patient demographics and characteristics

**Figure 4:**
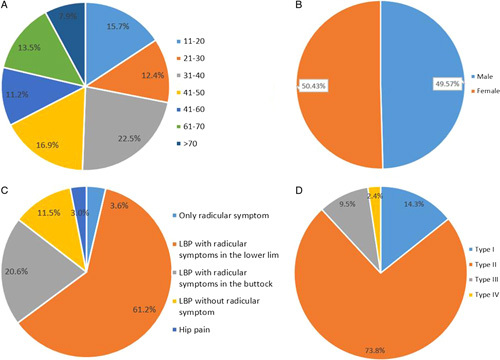
Patient demographics and characteristics (A) age; (B) sex; (C) symptoms; (D) Castellvi types. LBP, low back pain.

Of the 419 patients with Bertolotti’s syndrome, age was reported for 268 patients, with a mean age of 47.7 years (range, 12–80). Among them, 169 patients were included in six case series, with only their mean ages provided, and the specific age of the remaining 89 patients from the case reports was reported. Stratified by age group, 14 cases were aged between 11 and 20 years old (15.7%), 11 patients were aged between 21 and 30 years (12.4%), 20 patients were aged between 31 and 40 years (22.5%), 15 patients were aged between 41 and 50 years (16.9%), 10 patients were aged between 51 and 60 years (11.2%), 12 patients were aged between 61 and 70 years (13.5%), and 7 patients were older than 70 years (7.9%) in the case reports (Fig. 4 A).

Sex data was reported for 234 cases, comprising 116 males and 118 females, suggesting a male-to-female ratio of 1.02:1 (Fig. 4 B).

A total of 165 patients presented with LBP with/without radiculopathy or hip pain without radiculopathy (159 patients) or presented with only radiculopathy (6 patients). Of the 159 patients (96.4%), 101 had LBP radiating to the lower limbs, 34 had LBP radiating to the buttocks, 19 had LBP without radicular symptoms, and 5 had hip pain without radicular symptoms. Six patients had only radicular symptoms in the lower limbs or buttocks with no hip pain or LBP (Fig. 4 C).

The symptom durations were described for 143 patients, with the mean duration being 41.4 months (range, 2 months–16 years). The case with the longest symptom duration was a 56-year-old female patient who suffered low back and right upper buttock pain for 16 years before being diagnosed with Bertolotti’s syndrome based on imaging results.

The specific Castellvi types were reported in 262 cases. Type II was the most common type, which was reported in 196 cases (74.8%). Among the 196 patients, 73 patients were diagnosed with type IIa and 43 with type IIb; the remaining had no description of the laterality of the lesion. Type I occurred in 36 cases (13.7%), of which 15 were type Ia and 1 was type Ib; the Castellvi type was ambiguous in the remaining 20 cases. Of the 262 cases, 24 (9.2%) had type III, of which 20 were type IIIa and four were type IIIb. Type IV was only reported in 6 cases (Fig. 4 D).

Five studies reported the incidence of Bertolotti’s syndrome in LBP populations, which ranged from 4.6 to 10.6%. In the 15 414 patients with LBP included in these studies, LSTV were identified in 1341 patients, and the overall morbidity was 8.7%. Among the five studies, the study with the largest sample size investigated the radiographic data of 820 consecutive patients with symptoms related to the lumbosacral spine and found that 877 (10.6%) of the patients had Bertolotti’s syndrome.

Regarding comorbid spinal diseases, disc degeneration (bulge/herniation) was reported in 116 cases, L5/S1 degeneration in 88 cases, and L4/5 degeneration in 47 cases. Among the 116 cases, herniation was reported in 71 cases. In 186 cases reported in the analyzed studies, the incidence was 62.4%. Moreover, scoliosis was identified in 36 cases, and spina bifida was reported in 2 cases.

### Treatment strategy and outcomes

In 31 of the 419 cases (7.4%), the treatments were not adequately described since several studies focused on the diagnostic techniques for Bertolotti’s syndrome.

Conservative treatments, including (in various combinations) spine manipulations (*n*=19), physical therapies (*n*=15), nonsteroidal anti-inflammatory drugs (*n*=23), functional exercises (*n*=3), and corticosteroids (*n*=4), were provided to 54 patients.

Minimally invasive interventional therapies including local corticosteroid injections (*n*=47) and radiofrequency ablations (*n*=22) were provided to 69 patients.

A total of 265 patients underwent surgical treatments; the posterior approach was used in 260 cases, and the anterior approach was used in 5 cases. The following surgical techniques were performed (in various combinations): resection of the pseudoarticulation (*n*=221), removal of bone osteophytes (*n*=9), and fusion (*n*=35). The endoscopic technique was used in 57 cases; among them 51 cases underwent unilateral biportal endoscopy (UBE).

In these 265 patients, 227 (85.7%) received diagnostic local injections preoperatively, which provided transient symptom relief. The symptoms of five patients did not resolve after the preoperative injections, and one patient declined spinal injection due to steroid allergy. The symptoms of 13 patients did not resolve postoperatively, and 6 patients underwent revision surgeries. Reported surgical complications included new but temporary radicular pain (*n*=3), temporary weakness (*n*=1), haematoma (*n*=2), perirenal fluid collection (*n*=1), temporary paraesthesia (*n*=1), permanent new postoperative paraesthesia at the last follow-up (*n*=1), and genitofemoral nerve neurapraxia (*n*=1).

### Risk of bias and methodological quality

Based on the MINORS criteria, the mean score for the included studies was 4.16±3.95 points (range, 1−21). Only two of the included studies had a control group and used 12 items of the MINORS criteria, and the highest score was 21; the remaining studies did not have a control group and used only the first 8 items of the MINORS criteria. The highest score was 14.

### Research hotspots and frontiers for the field

**Figure 5:**
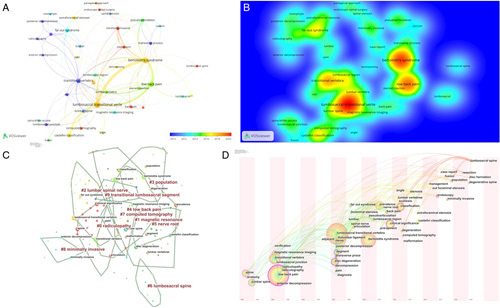
(A) Keywords co-occurrence network of publications; (B) Keywords density visualization map of publications; (C) The keywords co-authorship network; (D) The keywords timeline view of publications.

Figure 5 A demonstrates the keywords network map, which shows the research hotspots in this field. Besides “Bertolotti’s syndrome,” the other high-frequency keywords that were strongly associated with each other included “lumbosacral transitional vertebrae,” “low back pain,” “transitional vertebra,” “Castellvi classification,” and “far-out syndrome,” which coincided on the hotspots map (Fig. 5 B). All identified keywords were divided into 10 clusters (#0 radiculopathy, #1 magnetic resonance, #2 lumbar spinal nerve, #3 population, #4 LBP, #5 nerve root, #6 lumbosacral spine, #7 computed tomography, #8 minimally invasive, and #9 transitional lumbosacral segment), and the cluster name was refined according to the keywords in each cluster (Fig. 5 C). These clusters covered main research areas in the field of chordoma. Figure 5 D demonstrates the keywords timeline view of publications. A keyword burst list generated by Citespace that demonstrates the burst intensity and the year in which the burst began or ended is provided in Table 2. Over the 20-year observation period, the research focus in the early years was on the anatomy of the involved body parts (lumbosacral junction, transitional vertebra, transverse process, and iliolumbar ligament). Table 3 showed the top 10 keywords with the strongest citation bursts. In the middle years, the research focus shifted to the clinical characteristics of Bertolotti’s syndrome (far-out syndrome, foraminal stenosis, and scoliosis). In recent years, research on minimally invasive surgical techniques, prevalence, image classification, and disc degeneration, which are the main research focus of Bertolotti’s syndrome, has progressed remarkably, and these areas may be the future research directions for Bertolotti’s syndrome.

**Table 2:** Treatment strategy.

Treatment	Patients (*N*)
Conservative treatments	
Manipulations	19
Physical therapies	15
Nonsteroidal anti-inflammatory drugs	25
Functional exercises	3
Corticosteroids	4
Minimally invasive interventional therapies	
Local corticosteroid injections	47
Radiofrequency ablations	22
Surgical treatments	
Resection of the pseudoarticulation	221
Decompression of the bone osteophyte	9
Fusion	35
Endoscopic technique	57

**Table 3:**
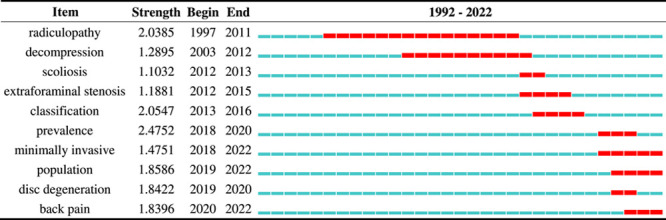
Top 10 keywords with the strongest citation bursts.

## Discussion

To the best of our knowledge, there is currently no consensus on the treatment of Bertolotti’s syndrome, and this study is the first bibliometric and systematic assessment of all scientific publications on Bertolotti’s syndrome, which included 118 publications. Based on the review of scientific literature, we aimed to answer the following two questions: (1) What is the prevalence, clinical characteristics, and management strategies of Bertolotti’s syndrome? and (2) What is the overall knowledge structure of Bertolotti’s syndrome, what are the global trends in research development, and what are the insights in research advancements.

The increase in the number of annual publications in this field has shown an overall steady growth in the past decade. According to the analysis based on country of publication, the USA, Japan, and China were the top three contributing countries. These countries have made significant efforts in research and contributed the most publications in the past decade. Regarding the journal of publication, Spine had the most publications, followed by Cureus J Med Sci, Asian Spine J, and Eur Spine J. Regarding citation analysis, there was a lack of high-level evidence-based information in the most cited articles. The clinical management of Bertolotti’s syndrome is still an intractable problem. Summarizing the findings from these existing studies based on excellent review and a consensus could provide constructive guidance for clinical decisions, which may help to resolve this conundrum.

In the early stage of the keyword co-citation analysis, we identified that there were active citations on lumbosacral junction, transitional vertebra, and transverse process. The results indicated that its anatomical characteristics had attracted the most attention in the early stage of the last decade because the pathogenesis of the disease was not well understood^30–39^. Recently, studies have increasingly focused on minimally invasive surgical techniques, image classification, and disc degeneration.

Regarding image classification, Castellvi classifications are the most widely used, and Bertolotti’s syndrome is classified into four types based on the relationship between the transverse process and the unilateral or bilateral sacrum. However, the credibility of the Castellvi classification in identifying morphological abnormalities is poor, with a sensitivity of 76–84% and an accuracy of only 53–58%^40^. In addition, Castellvi classifications do not consider the effect of the migrating vertebrae on the normal biomechanics of the intervertebral disc. Santavirta *et al.* ^29^ classified Bertolotti’s syndrome into five types, which are easily distinguishable than the Castellvi classifications. Using sagittal MRI, O’Driscoll *et al.* ^28^ classified Bertolotti’s syndrome into four types based on the morphology of the S1–S2 disc and the extent of lumbarization of the S1 segment, with a correlation between O’Driscoll type 4 and Castellvi type III or type IV. The Onyiuke Grading Scale, a novel grading system, classifies Bertolotti’s syndrome into four types according to the location, severity, and characteristics of the pain, with an extensive focus on clinical symptoms and less consideration of imaging outcomes^41^.

Computed tomography (CT) and MRI can provide more accurate diagnosis and classifications compared to that provided by traditional radiology while providing additional information such as intervertebral disc and nerve root pathologies. Despite the advantages of CT in assessing bone anatomy, the assessment of the accuracy of MRI is more common in the literature than that of CT. MRI can be more than 80% accurate in diagnosing Bertolotti’s syndrome^25,42,49^. The use of bone scintigraphy, such as SPET/CT and PET/CT, assists physicians with options for evaluating the possible sources of pain in patients with Bertolotti’s syndrome. EOS imaging is a relatively new radiographic system that provides accurate three-dimensional images of spinal anatomy and only emits low doses of radiation, which is valuable in differential diagnosis and classification^44^. Classification and imaging are inextricably linked in guiding treatment and for management. Due to advances in imaging technology, accurate counting of vertebral segments is a prerequisite for new classifications. Since many patients have a combination of disc herniation and phyletic variant spines, classification of Bertolotti’s syndrome should not only be important for the above-mentioned factors but should also guide treatment (especially the development of surgical approaches) and prognosis at the expense of some simplicity is inevitable.

Conservative treatment is usually the first choice of treatment for Bertolotti’s syndrome, and mostly includes manipulations, physical therapies, nonsteroidal anti-inflammatory drugs, functional exercises, and corticosteroids, of which none has been proven effective. When conservative symptomatic interventions are insufficiently effective or when there is progression of clinical symptoms, surgery is usually indicated. In our review, 68.3% of the patients received surgical treatment, with the posterior approach being the most used approach. Minimally invasive surgical techniques for only the pathological joint minimizes trauma to the posterior muscles and ligamentous structures, reduces the occurrence of postoperative back pain, and helps patients recover faster after surgery^30,45^. Minimally invasive surgery mainly includes microscopic decompression and endoscopic surgery; both surgical approaches have good treatment outcomes^46,47^. Meanwhile, with the improvement of endoscopic surgical techniques and the development of surgical instruments, the use of the full-endoscopic surgical approach as a treatment strategy is gradually increasing. UBE is an essential approach for endoscopic surgery. UBE is easier to control than controlling the single-channel approach and significantly improves the visualization of the surgical field, allowing for precise manipulation of the pathological site with a clear and magnified endoscopic view to achieve minimal soft tissue debridement and bleeding^30,48,49^. However, it should also be noted that the learning curve for endoscopic surgery is steep compared with that for microsurgery and requires sufficient experience in macroscopic surgery. Minimally invasive surgery causes some postoperative complications, such as haematoma, L5 radiculopathy, abdominal pain, incomplete decompression, and artery injury^30,50,51^. In addition, some studies have reported that radiofrequency ablation is used to relieve pain symptom, but it is difficult to evaluate its efficacy.

Bertolotti’s syndrome refers to a condition that shows an association of LBP with LSTV. It was previously known worldwide that disc and facet degenerations, which present as only LSTV-related degenerative findings, usually occur with increasing age. Subsequent studies have found a correlation between disc degeneration and LSTV, with three or more intervertebral disc levels being affected with the occurrence of LSTV, especially in young adults in whom the presence of LSTV significantly increases the incidence of disc degeneration^52–54^. Moreover, numerous studies have confirmed that discs above the transitional vertebra are more prone to early degeneration, while discs between the transitional vertebra and the sacrum are less prone to degeneration^7,12,16^. The mechanism may be explained by hypermobility, abnormal torque, altered mechanical stress, and weak iliolumbar ligament of the lumbosacral transitional vertebra^9,12,14^. In addition, different types of LSTV are associated with intervertebral disc degeneration. Cheng *et al.* ^54^ found that Castellvi type III and IV LSTVs were important risk factors for cranial disc (L4/5) degeneration, whereas type I LSTV was not significantly associated with disc degeneration. Farshad *et al.* ^53^ found that disc degeneration at the transitional level was most common in patients with type II LSTV and suggested that type I LSTV was not associated with disc degeneration. However, Apaydin *et al.* ^55^ found that disc degeneration at the transitional level was most common in young men with type I LSTV. The occurrence of LSTV closely correlates with disc degeneration, and surgeons are required to consider these factors, which could influence the selection of spinal levels for surgery and the formulation of surgical plans.

We conducted a comprehensive and objective search of articles published on Bertolotti’s syndrome to obtain reliable results and provided some insights into its clinical characteristics, overall knowledge structure, and global trends in research development. However, this study has limitations. First, only English articles were included, and unpublished and non-English articles were excluded. Second, bibliometric data changes with time, and delays in indexing of this study can lead to partial changes in the results. Third, the number of articles included in this study was relatively small, which can lead to bias in the interpretation of the analyzed results.

## Conclusions

This study showed an upward trend with a steady increase in the number of publications during the past two decades, which demonstrates researchers’ increased interest in Bertolotti’s syndrome. Our results showed a significant prevalence of Bertolotti’s syndrome in patients with LBP and a long duration of symptoms before treatment initiation. Moreover, the mean age of the patients in all the reported cases was 47.7 years, indicating that Bertolotti’s syndrome is an underdiagnosed cause of LBP, especially in young patients. Fortunately, stepwise therapy tended toward normalization, and a variety of surgical or non-surgical treatments are available. Surgical treatments were commonly used for patients after conservative treatment fails. With the progression in surgical technology, our study showed that minimally invasive surgical techniques continue to evolve. The association of Bertolotti’s syndrome with disc degeneration is another hotspot study area in recent years. The mechanism by which spinal biomechanical abnormalities occur secondary to LSTV remains unclear, which might be the future direction of research in this field. Randomized studies or guidelines on the treatment of Bertolotti’s syndrome are still lacking. The summarized clinical data and common trends established in our study might improve the understanding and management of Bertolotti’s syndrome.

## Ethical approval

IRB approval was not needed as this is a meta-analysis of published data; however, PROSPERO registration was done.

## Consent

Not applicable.

## Sources of funding

This research was supported by National Natural Science Fundation (82171127).

## Author contribution

Y.-C.L., C.-L.S.: conceptualization, formal analysis, software. Z.-Y.F., Y.-C.Z.: data curation, software, writing—original draft; writing—review and editing. Y.-X.H., B.-J.C., Y.J., S.-Y.Y., Z.T.: methodology, writing—review and editing; S.-Y.S., J.-P.W., B.-Y.L., L.Z., D.W., K.-L.W., H.-F.P., Y.-H.L.: software; supervision; validation; visualization, writing—review and editing.

## Conflicts of interest disclosure

We declare that we have no competing interests.

## Guarantor

Prof. Ye-Hai Liu.

## Availability of data and materials

All supporting data are available upon request to the authors.

## Provenance and peer review

Not commissioned, externally peer-reviewed.
